# Supplementary figures and images for: Antimicrobial resistance among farming communities in Wakiso District, Central Uganda: A knowledge, awareness and practice study
Source: PLoS One. 2023 Jun 2;18(6):e0284822. doi: 10.1371/journal.pone.0284822 (PMC10237438; doi:10.1371/journal.pone.0284822)

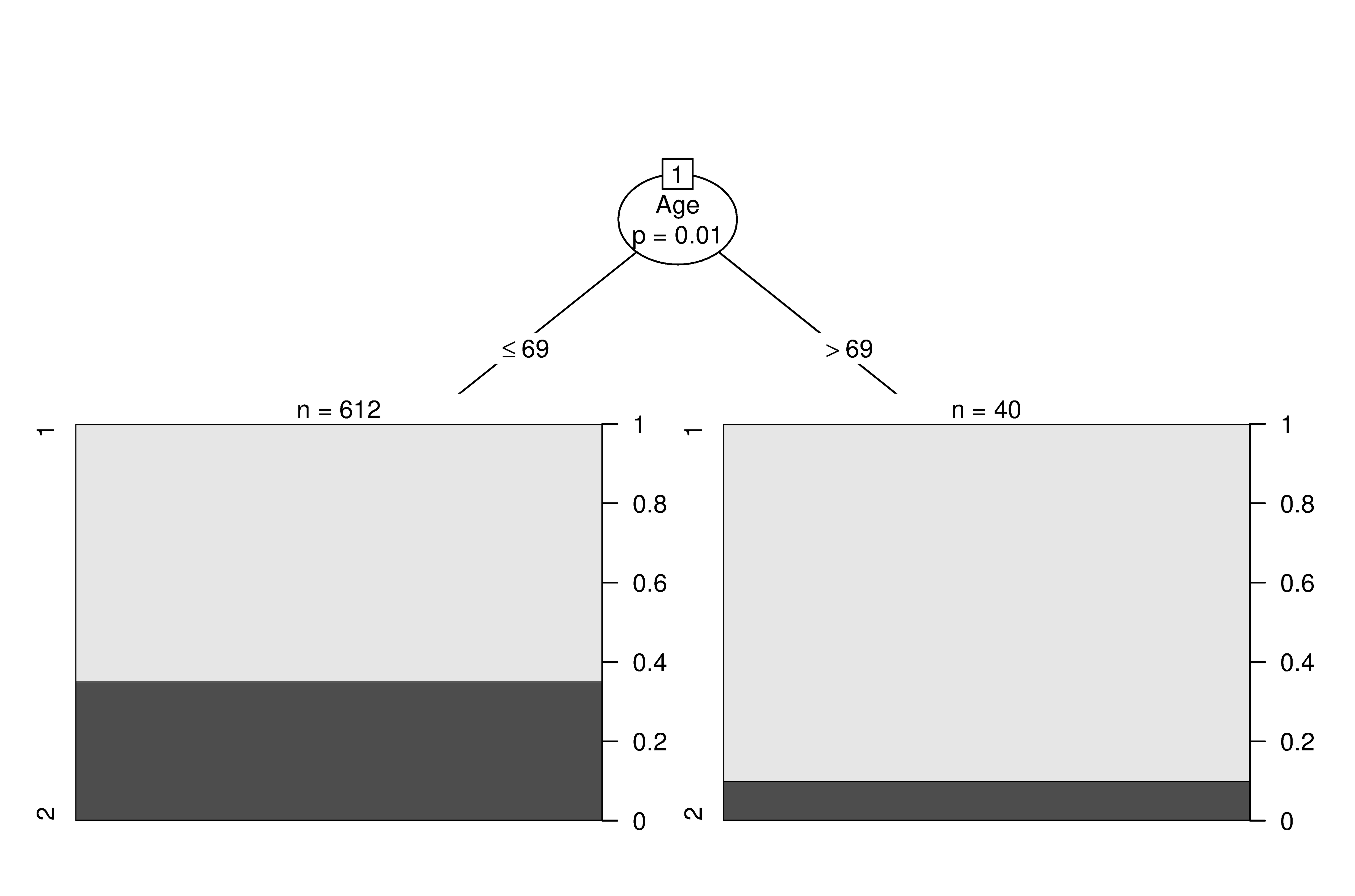

Supplement: S1 Fig — (TIF) [file pone.0284822.s001.tif]
