# Supplementary material for: Antimicrobial resistance among farming communities in Wakiso District, Central Uganda: A knowledge, awareness and practice study
Source: PLoS One. 2023 Jun 2;18(6):e0284822. doi: 10.1371/journal.pone.0284822 (PMC10237438; doi:10.1371/journal.pone.0284822)
Supplement: S1 File — This questionnaire was administered by interviewer in English or Luganda. (PDF) [file pone.0284822.s003.pdf]

S1: Questionnaire assessing Antimicrobial Drug residues in the food chain and antimicrobial resistance in Wakiso district

SECTION A: DEMOGRAPHIC CHARACTERISTICS

	Date:	Respondent ID
	Time Start …………<br><br>End …………	Respondent's contact:
	GPS location<br><br>Northings<br><br>Eastings	Town : ………………………………………………<br><br>Tc/Subcounty:<br>………………………………………<br>Parish<br>..<br><br>Village: ………………………………………
A1	What is your age in complete years?	………………………………
A2	What is the sex of participant?	1. Male<br>2. Female
A3	What is your marital status?	1. Married<br>2. Co-habiting<br>3. Divorced or separated<br>4. Widowed<br>5. Never married
A4	What is your highest level of education?	1. None<br>2. Primary<br>3. O' Level<br>4. A' Level<br>5. Technical/Vocational<br>6. University degree
A5	Which tribe do you belong to?	1. Muganda<br>2. Munyankole<br>3. Munyoro<br>4. Mukiga<br>5. Musongola<br>6. Other (specify) …………………………………
A6	What are the commonly used medications by you and your household members?	1. Antibiotics e.g. penicillin, tetracyclines and co-trimoxazole<br>2. Antimalarials<br>3. Antipyretics (pain killers)

		4. Other antimicrobials (specify) ....
A7	Of those mentioned above which ones do you see are no longer effective in treating you or your family member?	
A8	What is the main source of income for the household?	
A9	What is the main occupation of the household head?	1. Unemployed<br>2. Self-employed<br>3. Civil servant<br>4. Other (specify)<br>……………………………………
A9.1	What is your average monthly income for your household (continuous) (in Uganda shillings)	
A10	Main animal species kept (and their numbers)	1. Bovine (……………………..)<br>2. Caprine (…………………....)<br>3. Ovine (……………………..)<br>4. Swine (……………………..)<br>5. Equine (……………………..)<br>6. Others specify (……………..)
A11	Which food plants do you grow?	

SECTION B: KNOWLEDGE OF ANTIMICROBIALS AND ANTIMICROBIAL RESISTANCE

B1	Do you know what antimicrobials are?	1. Yes, can describe<br>2. No, can't describe
B2	Do you know what antibiotics are?	1. Yes, can describe<br>2. No,
B3	Do you have any antimicrobials at home today?	1. Yes<br>2. No … Skip to C3
B4.1	Can I please have a look at them? (For animals and crops)	Name of medicine:<br>Route of administration:<br>Dosage:
B4.2	Can I please have a look at them? (For human beings)	Name of medicine:<br>Route of administration:<br>Dosage:
B5	When do you think you should stop taking antibiotics once you've begun treatment?	1. When the medicine I bought is half/quarter done<br>2. When the medicine (full dose) is over<br>3. When I feel better<br>4. When I get reactions from it<br>5. Other(specify) …………………………….<br>99. Don't know
B6	Do you think this statement is 'true' or 'false'?<br>***"It's okay to use antibiotics that were given to a friend or family member, as long as they were used to treat the same illness"***	1. True<br>2. False<br>99. Don't know
B7	Do you think this statement is 'true' or 'false'?<br>***"It's okay to buy the same antibiotics, or request these from a doctor, if you're sick and they helped you get better when you had the same symptoms before"***	1. True<br>2. False (don't answer B10)<br>99. Don't know (don't answer B10)

B8	Do you think these conditions can be treated with antibiotics?	1. HIV/AIDS<br>2. Urinary tract infection (UTI)<br>3. Diarrhoea<br>4. Cold and flu<br>5. Fever<br>6. Malaria<br>7. Measles<br>8. Skin or wound infection<br>9. Sore throat<br>10. Body aches<br>11. Headaches<br>12. Any other unmentioned? (specify)
B9	Have you heard of any of the following?	1. Antibiotic resistance
		2. Drug residues
		3. Superbugs
		4. Drug resistance
		5. Antibiotic-resistant bacteria/bacteria
B10	If true to B7, where did you hear about the term? (Multiple options allowed)	1. Doctor or nurse<br>2. Pharmacist<br>3. Family member or friend<br>4. Media<br>5. Specific campaign<br>6. Veterinarian<br>7. Other(specify) …………<br>99. Can't remember
B11	**Please indicate whether you think the following statements are 'true' or 'false'.**	
B11.1	Antibiotic resistance occurs when your body becomes resistant to antibiotics and they no longer work as well.	1. True<br>2. False<br>99. Don't know
B11.2	Many infections are becoming increasingly resistant to treatment by antibiotics.	1. True<br>2. False<br>99. Don't know
B11.3	If bacteria are resistant to antibiotics, it can be very difficult or impossible to treat the infections they cause.	1. True<br>2. False<br>99. Don't know
B11.4	Antibiotic resistance is an issue that could affect me or my family.	1. True<br>2. False<br>99. Don't know
B11.5	Antibiotic resistance is an issue in other countries but not in our country.	1. True<br>2. False<br>99. Don't know
B11.6	Antibiotic resistance is only a problem for people who take antibiotics regularly.	1. True<br>2. False

		99. Don’t know
B11.7	Bacteria which are resistant to antibiotics can be spread from person to person.	1. True<br>2. False<br>99. Don’t know
B11.8	Antibiotic-resistant infections could make medical procedures like surgery, organ transplants and cancer treatment much more dangerous	1. True<br>2. False<br>99. Don’t know

SECTION C: On the scale shown, how much do you agree the following actions would help address the problem of antibiotic resistance?

		**Strongly Agree** (5)	**Agree Slightly (4)**	**Neither agree nor disagree (3)**	**Disagree Slightly (2)**	**Disagree Strongly (1)**
C12.1	People should use antibiotics only when they are prescribed by a health practitioner.					
C12.2	Farmers should give fewer antibiotics to food-producing animals.					
C12.3	People should not keep antibiotics and use them later for other illnesses.					
C12.4	Parents should make sure all of their children's vaccinations are up-to-date.					
C12.5	People should wash their hands regularly.					
C12.6	Doctors should only prescribe antibiotics when they are needed.					
C12.7	Governments should reward the development of new antibiotics.					
C12.8	Pharmaceutical companies should develop new antibiotics.					

SECTION D: ACCESS TO AND USE OF ANTIMICROBIALS IN ANIMALS

D1	Do you rear any animals in this household?	1. Yes<br>2. No ……………………… ***Skip to the next section***
D2	What animals do you own in this household?<br><br>***(Mark all that apply)***	1. Poultry<br>2. Pig<br>3. Cattle<br>4. Goat / sheep<br>5. Dog / cat<br>6. Others (specify) ……………………………………………….
D3	What drugs do you commonly use among animals in your household?<br><br>***(Mark all that apply)***<br><br>***Ask to find out if they know the name of the drug. If not, ask to see the bottle / container / package if available to identify the name of the drug. If more than 3 drugs are being used, note the 3 most used***	Poultry<br><br>1……………………………………………………<br><br>2. ……………………………………………………<br><br>3. ……………………………………………………<br>4. None<br>5. Other (specify) …………………………………………………….<br>6. Don't know<br><br>Pig<br><br>1……………………………………………………<br><br>2. ……………………………………………………<br><br>3. ……………………………………………………<br>4. None

		5. Other(specify) …………………………………………………………. 6. Don't know<br><br>Goat / sheep<br><br>1……………………………………………………… ………<br><br>2. ……………………………………………………… …<br><br>3. ………………………………………………………<br>4. None<br>5. Other(specify) ……………………………………………………… ….<br>6. Don't know<br><br>Cattle<br><br>1……………………………………………………… ………<br><br>2. ……………………………………………………… …<br><br>3. ……………………………………………………… …<br>4. None<br>5. Other(specify) ……………………………………………………… ….<br>6. Don't know<br><br>Dog / cat<br>1……………………………………………………… …<br><br>2. ……………………………………………………… …

		3. ………………………………………………………<br>4. None<br>5. Other(specify) ……………………………………………………<br>6. Don't know<br><br>*If no drugs are used among animals in the household, skip to the next section*
D4	Why do you normally use these drugs in your household?<br><br>***(Mark all that apply)***	1. To treat sick animals<br>2. To prevent sick animals becoming sick<br>3. To fatten / increase growth of the animals<br>4. To kills ticks and other pests<br>5. Other (specify) ………………………………………………
D5	***Skip this question if option 1 (To treat sick animals) was mentioned in question D4 above.***<br><br>When administering these drugs to treat sick animal, which animals do you normally give them to?<br><br>***(Mark all that apply)***	1. Only sick animals<br>2. All animals of the same species<br>3. All animals (different species)<br>4. Other(specify) …………………..…………………………
D6	How do you normally access these drugs?<br><br>***(Mark all that apply)***	1. Veterinary worker<br>2. Veterinary drug shop<br>3. Human pharmacy / drug shop<br>4. Market<br>5. Other farmers<br>6. Other (specify) ………………………………..…………
D7	***Ask this question if mentioned option 3 (human pharmacy / drug shop) in question D6 above***	1. Easily available<br>2. Cheaper<br>3. They are more effective<br>4. Other (specify)……………………………………………..

	Why do you use human drugs for treating your animals?	
D8	Do you normally get advice about how to use drugs among animals?	1. Yes<br>2. No (***Skip to D10)***…………………………….……………….
D9	From which avenues do you normally get advice on use of drugs among animals?<br><br>***(Mark all that apply)***	1. Veterinary worker<br>2. Human health worker (including pharmacy / drug shop)<br>3. Other farmers<br>4. Package label<br>6. Other(specify) ……………………………………
D10	For how long do you normally use these drugs?<br><br>***(Mark all that apply)***	1. As recommended by the provider<br>2. Until the animal is cured<br>3. Until the package is empty<br>4. Once<br>5. As long as I can afford<br>6. Other (specify) …………………………………….
D11	Who normally administers the drugs?	1. Member of the household<br>2. Owner of the animal<br>2. Veterinary worker<br>3. Animal attendant<br>3. Other(specify) ……………………………………
D12	How are the drugs normally administered?<br><br>***(Mark all that apply)***	1. Orally<br>2. Injection<br>3. With water<br>4. With feeds<br>5. Other(specify) …………………………………..
D13	Do you sell or consume animal products (milk, meat or eggs) from animals that were recently treated with drugs?	1. Yes (***Skip to D15)***…………………….………… 2. No
D14	For how long would you wait between treatment of the animal and consumption of its product?	1. Manufacturers recommendation<br>2. As per advice of a veterinary worker<br>3. As per human health workers advice<br>4. My own judgement<br>5. Other(specify) …………………………………….

D15	What do you normally do with animal drugs that have expired, empty bottles and sachets?<br><br>***(Mark all that apply)***	1. Collected from residence<br>2. Deposited in communal bins and collected<br>3. Placed in a rubbish pit next to house<br>4. Placed in a communal rubbish pit<br>5. Burned<br>6. Thrown in a drain/open area<br>7. Thrown in a pit latrine<br>8. Other (specify) ……………………………………..
D16	What are some of the health effects of antimicrobial use in humans and animals	1. Diseases such as a cute respiratory infections and tonsillitis<br>2. Increased treatment costs<br>3. Reduced productivity<br>4. Others specify
